# Psychiatric Symptoms and Parental Stress in Children and Adolescents With Epilepsy

**DOI:** 10.3389/fneur.2021.778410

**Published:** 2021-12-08

**Authors:** Francesca Felicia Operto, Grazia Maria Giovanna Pastorino, Federica Pippa, Chiara Padovano, Valentina Vivenzio, Chiara Scuoppo, Ilaria Pistola, Giangennaro Coppola

**Affiliations:** Child Neuropsychiatry Unit, Department of Medicine, Surgery and Dentistry, University of Salerno, Salerno, Italy

**Keywords:** epilepsy, children, behavioral problems, emotional problems, parental stress

## Abstract

**Introduction:** The aim of this study was to identify the presence of emotional and behavioral symptoms in children and adolescents with epilepsy, to measure the stress levels in their parents, and to determine if and how parental stress was linked to emotional and behavioral symptoms of their children.

**Methods:** We conducted a cross-sectional observational study including 103 children and adolescents with different form of epilepsy and 93 sex-/age-matched controls. Parental stress and emotional and behavioral symptoms were assessed through two standardized questionnaires: the Parenting Stress Index (PSI) and the Child Behavior Checklist (CBCL), respectively. We also considered the following variables: age, sex, maternal education level, family history of psychiatric disorders, duration of epilepsy, seizure frequency, seizure type, and number of antiseizure medications.

**Results:** The statistical comparison showed that the epilepsy group obtained significantly higher scores than controls in almost all the CBCL and the PSI scales (*p* < 0.05). The correlation analysis revealed a significant relationship between the PSI Total Stress scale and the following CBCL scales: total problems, internalizing problems, and externalizing problems (*p* < 0.05). An earlier age of seizure onset was related to a greater presence of externalizing problems, total problems, and total stress (*p* < 0.05).

**Conclusion:** In the epilepsy group, we found higher levels of parental stress and higher presence of emotional and behavioral symptoms compared to controls, mainly represented by internalizing problems (anxiety and depression symptoms). Therefore, it is important to precociously detect these symptoms and monitor them over time, in order to prevent psychiatric problems. In addition, parents of children with epilepsy should be offered psychological support to cope with parental stress and to improve the relationship with their children.

## Introduction

Epilepsy is one of the most frequent chronic neurological conditions in pediatric population, with the highest incidence in the first year of life. Worldwide, over 10 million of children and adolescents under the age of 15 years suffer from epilepsy, accounting for about a quarter of the total individuals with epilepsy (1).

Children and adolescents with epilepsy exhibit emotional and behavioral problems more often than children in the general population (2–6), including depression, anxiety, psychosis, attention, and behavioral problems, as a result of both the psychosocial (unpredictability and distressing nature of the seizures, social stigma associated with epilepsy, and overprotective parental behavior) and clinical factors (etiology, age at onset of epilepsy, frequency, and severity of seizures) (7–10).

The emotional and behavioral problems in children with epilepsy may also be influenced by family factors such as socioeconomic status or psychiatric conditions in other family members (11, 12).

Several epidemiological studies, focusing on the prevalence of psychopathological symptoms in pediatric epilepsy, documented that children with epilepsy present an estimated overall risk of 21–60% for childhood psychopathology (7, 13). A review of Reilly et al. (4) reported the presence of depression in 12–14% of pediatric epilepsy in population-based studies. The authors suggested that significant variations in instruments and methods used to assess anxiety and depression in published studies could lead to variable results (4). With respect to the prevalence of anxiety in pediatric epilepsy, a study by Williams et al. (14) reported mild-to-moderate symptoms of anxiety in 23% of patients (14). However, for both the anxiety and depression, the prevalence rates appear to be higher in young people with epilepsy than the general pediatric population and in children with other chronic medical conditions not involving the central nervous system (3, 15, 16).

A recent population-based study of young people with epilepsy aged between 0 and 17 years highlighted that 43% of the subjects showed psychiatric or neurodevelopmental comorbidities. More severe forms of epilepsy were more often associated with the risk of developing psychiatric comorbidities; even milder conditions were burdened by the presence of emotional and behavioral problems (17, 18).

Psychiatric and behavioral comorbidities in these children should not be attributed exclusively to the chronicity of the disease, but the presence of some specific epilepsy-related factors, including the underlying brain dysfunction, might be supposed (19).

The mechanisms underlying the development of psychiatric comorbidities in epilepsy are supposed to be multiple and complex. Although they have not been fully clarified, it is possible to advance several hypotheses: the first one is the presence of a genetic risk shared between epilepsy and psychiatric disorders that affects the development of common neural systems. The second one is that seizures themselves can lead to the construction of inadequate cortical networks, involving the limbic and frontocentral cortex (20). Moreover, it must be considered that epilepsy is more often associated with impairments of the cognitive profile, executive functions, social cognition, and learning skills that could lead to social and scholastic difficulties (21–25). Finally, social stigma can further contribute to increasing the emotional burden in young people with epilepsy (26). With respect to the role of antiseizure medications (ASMs), some drugs seem to have a higher tolerability profile than others; generally, the reduction of the seizures and a better control of the disease are associated with a more favorable emotional and behavioral profile (22, 27–30).

Psychiatric comorbidities and emotional and behavioral problems place a significant burden on patients and their families and complicate the clinical management of epilepsy (31).

Chronic diseases in children, such as diabetes, asthma, and autism, can generate parental stress; therefore, having children with epilepsy, characterized by unpredictable crisis onset, can cause treatment-related stress in their parents (32, 33).

Our cross-sectional observational study aimed to evaluate the presence of emotional and behavioral symptoms in children and adolescents with epilepsy through a standardized neuropsychological assessment compared to sex-/age-matched controls. We also aimed to explore the correlation between these symptoms and epidemiological and clinical variables (sex, age, etiology of epilepsy, age at onset of epilepsy, epilepsy duration, seizure type, seizure frequency, number of ASMs, and family history of psychiatric condition).

The secondary aim of this study was to measure the stress level in parents of children with epilepsy and to determine if and how parental stress is linked to emotional and behavioral symptoms of their children.

## Materials and Methods

### Study Design

We conducted a cross-sectional observational study that aimed to explore emotional and behavioral symptoms in young patients with epilepsy and the stress levels in their parents.

### Participants

We consecutively enrolled children and adolescents aged between 6 and 18 years, diagnosed with different types of epilepsy at the Child and Adolescent Neuropsychiatry Unit of the University of Salerno, from June 2019 to February 2021.

The diagnosis was made by two expert clinicians, according to the most recent classification of the International League Against Epilepsy (ILAE) (2017) (34), based on the electroencephalogram (EEG) findings and on the typical clinical features of the seizures. The MRI study supported the diagnosis if it was needed.

We also recruited a control group, homogeneous by sex, age, and socioeconomic status, among children and adolescents belonging to a screening project for learning difficulties. These subjects were healthy children without any presence of medical, neurological, and psychiatric conditions.

In all the patients of the control group, the diagnosis of epilepsy was excluded and all had a normal EEG.

Exclusion criteria in both the groups were the presence of additional neurological [cerebral palsy, moderate-to-severe intellectual disability according to the Diagnostic and Statistical Manual of Mental Disorders-5 (DSM-5) criteria, neurodegenerative diseases, or migraine], psychiatric (anxiety, depression, and psychosis), or other relevant medical conditions (endocrinological, metabolic, hepatic, cardiac, or renal disorders).

Two standardized neuropsychological questionnaires were administered to the parents of all the children by a single child neuropsychiatrist, evaluating emotional and behavioral problems of child and parental stress level.

We also recorded the following clinical variables: age at onset of epilepsy, disease duration, epileptic seizure frequency, lobe and side of epileptic seizure onset, and ASMs numbers.

A clear and detailed explanation about the purposes and the procedures of this study was provided to all the participants and their parents. Parents provided their informed consent in written form. The procedure was approved by the local ethics committee “Campania Sud,” according to the rules of good clinical practice, in keeping with the Declaration of Helsinki.

Table 1 shows the main sample characteristics.

**Table 1:** Demographic and clinical characteristics.

	**Epilepsy group**	**Control group**	**Statistics**
***N***	103	93	
**Sex**			
Male	60 (58%)	52 (56%)	χ^2^ = 0.109
Female	43 (42%)	41 (44%)	*p* = 0.741
Age in years (M ± SD)	12.54 ±3.87	11.84 ± 3.51	*U* = 4355.5
			*p* = 0.272
Maternal Education (years)	12.44 ± 6.72	13.84 ± 6.98	*U* = 4656
			*p* = 0.214
Familiar history of psychiatric disorders	19 (19%)	12 (13%)	χ^2^ = 1.127
			*p* = 0.288
**Epilepsy characteristics**			
Age at onset (M ± SD)	9.04 ±3.21		
Epilepsy duration in year (M ± SD)	3.30 ± 3.84		
**Etiology**			
Genetic	10 (10%)		
Symptomatic	12 (12%)		
Unknown	81 (78%)		
**Seizure frequency**			
Monthly	59 (57%)		
Weekly	20 (19%)		
Daily	8 (8%)		
Seizure free	16 (16%)		
**Seizure type**			
Focal	42 (42%)		
Generalized	26 (25%)		
Unknown	35 (33%)		
**Drug therapy**			
Mono	44 (43%)		
Poli	59 (57%)		
Number of ASMs (M ± SD)	1.18 ± 0.84		
MRI positive	12 (12%)		
-	6 cortical dysplasia 6 hypoxic-ischemic damage		

*N, sample size; M, mean; ASM, antiseizure medication*.

### Neuropsychological Assessment

#### Child Behavior Checklist for Ages 6–18 (CBCL/6–18)

The CBCL/6-18 (35) is a standardized questionnaire for parents that evaluate emotional, social, and behavioral problems in children aged between 6 and 18 years. The questionnaire consists of 113 questions, to which parents can answer with a Likert scale ranging from 0 to 2 (0 = not true, 1 = somewhat or sometimes true, and 2 = very true or often true). Raw scores are converted to T-scores, weighted by sex and age. It is possible to obtain the scores of three main scales (“internalizing problems,” “externalizing problems,” and “total problems”), six scales based on the DSM-IV (“affective problems,” “anxiety problems,” “somatic problems,” “attention deficit hyperactivity disorder (ADHD) problems,” “oppositional defiant problems,” and “conduct problems”), and eight empirically-based syndrome scales (“anxious/depressed,” “withdrawn/depressed,” “somatic complaints,” “social problems,” “attention problems,” “rule-breaking behavior,” and “aggressive behavior”) in which a T-score ≤ 64 indicates non-clinical symptoms, a T-score between 65 and 69 indicates a borderline range, and a T-score ≥ 70 indicates clinical symptoms.

#### Parenting Stress Index-Short Form (PSI-SF)

The PSI (36, 37) is a standardized questionnaire for parents that measure the level of stress in the dyad parent-child. The short form of PSI consists of 36 items, to which parents attribute a score on a Likert scale ranging from “5 = strongly agree” to “1 = strongly disagree.”

This self-report is organized in different subscales: parental distress (PD), parent-child dysfunctional interaction (P-CDI), and difficult child (DC), which evaluate the level of distress a caregiver is experiencing in his/her parental role, the satisfaction in the relationship with their own child, and, lastly, how difficult the management of the child is perceived to be.

The test also allows to evaluate the Total Stress (TS) scale. The TS is obtained by adding the relative scores of the three subscales (PD, P-CDI, and DC).

Raw scores are converted in age-weighted scores. A higher score suggests a higher stress level and a score above 85 indicates clinically significant parental stress.

#### Statistical Analysis

All the neuropsychological scores were expressed as mean ± SD. The percentage of participants scoring lower than the normal (<2 SD) was evaluated. In order to verify the data distribution, the Kolmogorov–Smirnov normality test was preliminarily performed. The presence of data not normally distributed forced us to employ non-parametric tests for our analysis. The comparison of proportions was made using the chi-squared test, whereas the comparison of the mean scores was performed using the Mann–Whitney *U* test (independent sample).

The two-tailed Spearman's rank correlation test was employed to evaluate the relationship between different variables. All the data were analyzed using Statistical Package for the Social Science (SPSS) software (version 25.0, SPSS Incorporation, Armonk, New York, USA) (IBM Corporation released, 2017); *p*-values ≤0.05 are considered as statistically significant.

## Results

### Sample Characteristics

**Figure 1:**
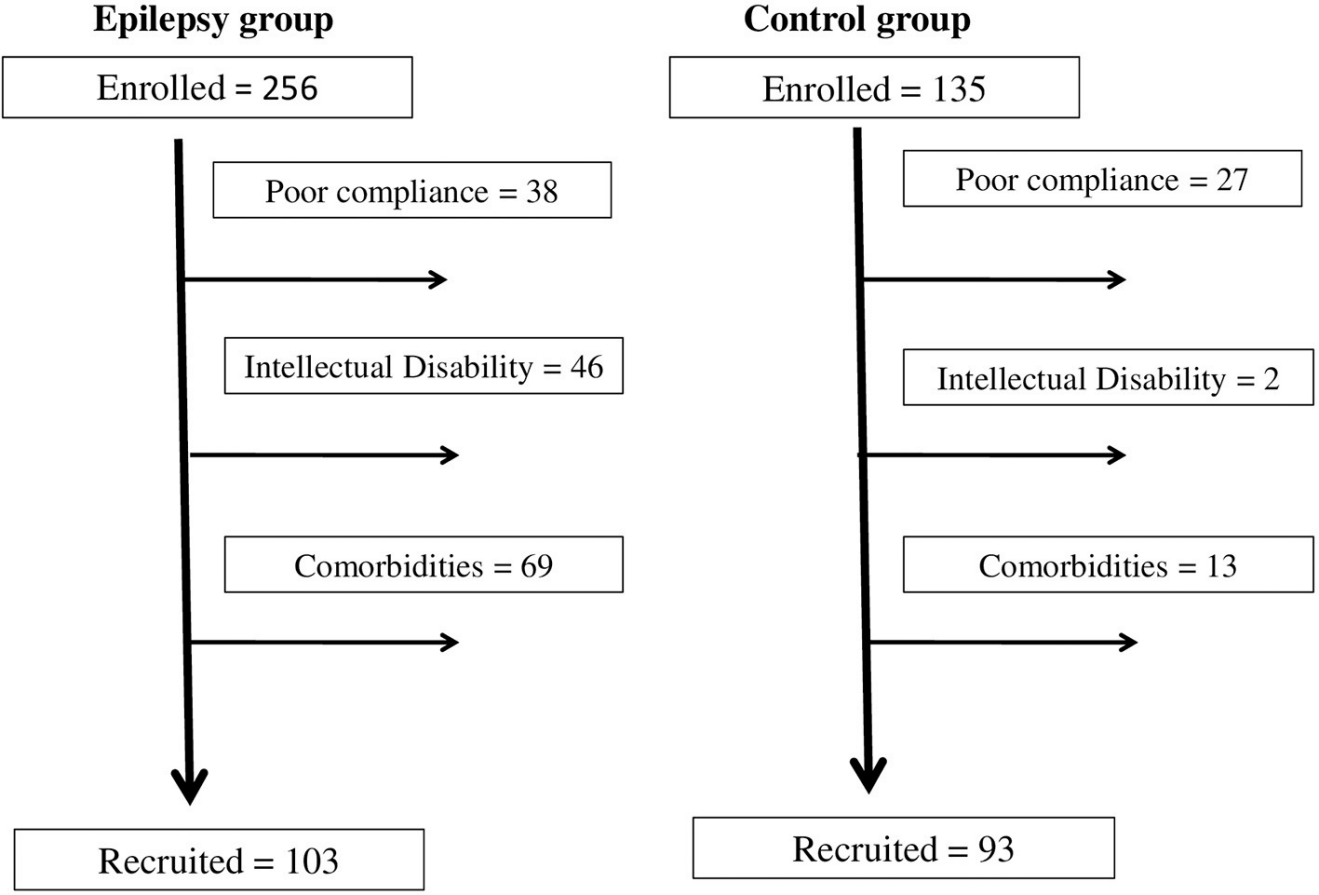
Recruitment flowchart.

We recruited 103 children (*n* = 38; age < 12 years) and adolescents (*n* = 65; age ≥ 12 years) diagnosed with epilepsy (mean age = 12.34 years, *SD* = 3.87 years) and 93 sex-/age-matched controls (mean age = 11.84 years, *SD* = 3.52 years) (Figure 1).

All the demographic and clinical characteristics of the participants such as age, sex, level of maternal education, seizure types, seizure frequency, age at onset of epilepsy, epilepsy duration, MRI findings, and ASMs number are shown in Table 1. The epilepsy group and the control group did not significantly differ in the main demographic characteristics (Table 1).

A family history of psychiatric disorders was found in 19% of the subjects with epilepsy. The mean age of epilepsy onset was 9.04 (±3.21) years, with a mean disease duration of 3.30 (±3.84) years. In most cases, the etiology of epilepsy was unknown; in 10%, we found a genetic mutation, in 6%, we found a cortical dysplasia, and in the remaining 6%, we found a hypoxic-ischemic damage. All the patients were in drug therapy with ASMs (43% monotherapy and 57% polytherapy).

Seizure type and frequency were shown in Table 1.

### Emotional and Behavioral Symptoms in the Epilepsy Group vs. the Control Group

Analyzing the CBCL Total Problems scale, we found that 25/103 (24%) of patients with epilepsy obtained a score higher than the norm (T-score ≥ 70), against 8/93 (9%) of controls.

Internalizing problems were present in 30/103 (29%) and externalizing problems were present in 20/103 (19%) of children and adolescents with epilepsy vs. 7/93 (7%) and 6/93 (6%) of the controls, respectively. The remaining clinical scores percentages are given in Table 2.

**Table 2:** Statistical comparison between the average scores of the different groups.

	**Epilepsy (m ± SD)**	**Control (m ± SD)**	**Mann-Witney**		**Percentage of subjects scoring** **in clinical range**	
			***U***	***P***	**Epilepsy**	**Control**
**CBCL**						
Anxiety/depression	62.27 ± 11.06	57.91 ± 7.37	3,808	**0.018**	29 (27%)	5 (5%)
Withdrawn/depressed	62.01 ± 10.19	56.32 ± 7.92	3,109	**0.000**	26 (25%)	5 (5%)
Somatic complaints	63.61 ± 11.23	57.97 ± 8.97	3,313	**0.000**	33 (32%)	13 (14%)
Social problems	62.57 ± 9.38	59.86 ± 9.53	3,861	**0.019**	22 (21%)	4 (4%)
Thought problems	60.28 ± 10.08	54.81 ± 6.74	3,168	**0.000**	24 (23%)	3 (3%)
Attention problems	63.56 ± 12.35	58.84 ± 10.64	3,591	**0.002**	26 (25%)	7 (7%)
Rule-breaking behavior	57.19 ± 8.52	55.10 ± 6.36	4263.5	0.181	14 (14%)	4 (4%)
Aggressive behavior	61.58 ± 13.08	54.98 ± 8.33	3061.5	**0.000**	16 (15%)	5 (5%)
Affective problems	61.68 ± 9.12	53.69 ± 8.06	1445.5	**0.000**	18 (17%)	3 (3%)
Anxiety problems	62.31 ± 7.82	54.28 ± 7.59	1,434	**0.000**	19 (18%)	4 (4%)
Somatic problems	60.91 ± 10.06	56.26 ± 9.50	2,289	**0.006**	24 (23%)	8 (9%)
ADHD problems	60.37 ± 8.82	54.56 ± 7.99	1,860	**0.000**	16 (16%)	4 (4%)
Oppositional-defiant problems	58.65 ± 8.46	53.43 ± 7.09	1908.5	**0.000**	9 (9%)	2 (2%)
Conduct problems	57.84 ± 9.65	52.77 ± 7.00	1,846	**0.000**	9 (9%)	0 (0%)
Internalizing problems	61.38 ± 13.20	56.56 ± 8.33	3564.5	**0.002**	30 (29%)	7 (7%)
Externalizing problems	57.78 ± 13.07	53.56 ± 8.67	3623.5	**0.003**	20 (19%)	6 (6%)
Total problems	61.99 ± 12.66	58.68 ± 10.00	3915.5	**0.027**	25 (24%)	8 (9%)
**PSI**						
PD	70.05 ± 27.41	34.55 ± 29.00	1787.5	**0.000**	46 (45%)	10 (11%)
P-CDI	79.08 ± 20.68	40.97 ± 26.68	1,365	**0.000**	59 (57%)	10 (11%)
DC	75.53 ± 24.01	39.61 ± 29.39	1,724	**0.000**	51 (49%)	11 (12%)
TS	75.83 ± 24.90	36.16 ± 26.98	1,432	**0.000**	52 (50%)	10 (10%)

*p < 0.05 is in bold*.

*m, mean; CBCL, Child Behavior Checklist; PSI, Parental Stress Index; TS, Total Stress; PD, parental distress; P-CDI, parent–child dysfunctional interaction; DC, difficult child; DR, defensive response*.

The mean scores comparison showed that the epilepsy group obtained significantly higher scores than controls in all the CBCL scales, with the exception of the Rule-Breaking Behavior scale (*p* < 0.05).

Table 2 shows all the neuropsychological mean scores for the CBCL in both the groups and the results of statistical comparison.

### Parental Stress in the Epilepsy Group vs. the Control Group

Analyzing the PSI TS scale, we found that 52/103 (50%) of parents of children with epilepsy obtained a score higher than the norm (T-score ≥ 85), against 3/93 (3%) of controls. The remaining clinical scores percentages are shown in Table 2.

The statistical comparison showed that the epilepsy group obtained significantly higher scores than controls in all the PSI scales (*p* < 0.05).

Table 2 shows all the neuropsychological mean scores for the PSI in both the groups and the results of the statistical comparison.

### Correlation Analysis

The correlation analysis revealed a statistically significant positive relationship between the PSI TS scale and the following CBCL scales: total problems, internalizing problems, and externalizing problems (*p* < 0.05).

A lower age of seizure onset was correlated with a higher presence of externalizing problems, total problems, and TS (*p* < 0.05).

We did not find statistically significant correlations among the other analyzed variables (age, sex, maternal education level, family history of psychiatric disorders, duration of epilepsy, seizure frequency, seizure type, and number of ASMs) and the CBCL and the PSI.

Table 3 shows the significant results of the correlation analysis.

**Table 3:** Correlation analysis.

	**PSI**	**CBCL**		
	**Total stress**	**Internalizing problems**	**Externalizing problems**	**Total problems**
Age at onset	*r =* −0.239		*r =* −0.231	*r =* −0.210
	***p =*** **0.015**		***p =*** **0.019**	***p =*** **0.034**
Internalizing	*r =* 0.227		*r =* 0.722	*r =* 0.856
problems	***p =*** **0.021**		***p =*** **0.000**	***p =*** **0.000**
Externalizing	*r =* −0.222	*r =* 0.722		*r =* −0.866
problems	***p =*** **0.024**	***p =*** **0.000**		***p =*** **0.000**
Total Problems	*r =* 0.199			
	***p =*** **0.044**			

*p < 0.05 is in bold*.

*CBCL, Child Behavior Checklist; PSI, Parental Stress Index*.

## Discussion and Conclusion

Previous studies already highlighted the presence of both the emotional and behavioral symptoms and psychiatric problems in pediatric populations with epilepsy (38, 39). In particular, a very recent meta-analysis by Scott et al. (40) showed that over 70% of children and adolescents with epilepsy had at least one psychological symptom, with a prevalence of anxiety of 18.9% and of depression of 13.5%, in pooled data; the estimated risk of ADHD was between 2.5 and 5.5 times higher in children and adolescents with epilepsy than in controls. Serra-Pinheiro et al. (9) reported a prevalence of 22% for mood disorders, 20.7% for anxiety, 26.8% for attention deficit, and 24.4% for disrupted disorders in a sample of children with epilepsy, while in another very recent work of Shehata and colleagues (41) on 80 children aged 6–13 years with idiopathic epilepsy the presence of depressive symptoms rose to 37.5%.

Our cross-sectional observational study aimed to evaluate the presence of emotional and behavioral symptoms in children and adolescents with epilepsy, through a standardized neuropsychological assessment, and compare them to sex-/age-matched controls. We also measured the stress level in their parents in order to determine if and how parental stress is linked to emotional and behavioral symptoms of the children.

From the analysis of the CBCL questionnaire administered to the parents, it emerged that children and adolescents with epilepsy (*n* = 103) compared to their peers (*n* = 93) had a significantly higher number of total emotional and behavioral problems (24 vs. 9%), with a slight prevalence of internalizing problems (29 vs. 7%) compared to externalizing problems (19 vs. 6%).

In the empirical CBCL scales, the most represented problems were somatic complaints (32%) and anxiety/depression (27%), followed by withdrawal/depression and attention problems. Thought problems and socialization problems were present in 23 and 21% of subjects, respectively. The presence of aggressive behavior was reported in 15% of cases and the presence of rule-breaking behavior was reported in 14% of cases (Table 2). The DSM-IV-oriented scales highlighted the presence of somatic problems (24%), anxiety problems (19%), affective problems (18%), ADHD problems (16%), oppositional defiant problems (9%), and conduct problems (9%) (Table 2).

The analysis of these results revealed a significant difference between the empirical CBCL scales compared to the DSM-IV-oriented scales. Therefore, we suggest that it would be useful to employ the DSM-IV-oriented scales instead of empirical scales, which contain elements clearly not belonging to the problem that the scale intends to evaluate.

From the statistical comparison with the control group, we found that children and adolescents with epilepsy presented significantly worse symptoms in all the emotional and behavioral areas, with the exception of the Rule-Breaking Behavior scale (Table 2).

This study extends and confirms the results of previous studies that explored emotional and behavioral symptoms through the CBCL in children with epilepsy.

In the study by Jones and colleagues (42), the authors found that children with recent onset epilepsy exhibited an elevated rate of the DSM-IV axis I disorders compared to controls. They showed significantly higher rates of depressive disorders (22.6 vs. 4%, *p* = 0.01), anxiety disorders (35.8 vs. 22%, *p* < 0.05), and ADHD (26.4 vs. 10%, *p* = 0.01). A subset of children with epilepsy (45%) exhibited these problems before the first recognized seizure, suggesting the potential influence of antecedent neurobiological factors that remain to be identified (42).

Our findings agree with Del Canto and colleagues (2018) (43). The authors highlighted emotional and behavioral problems in 50% of 159 children with epilepsy. Similarly, internalizing problems were more present than externalizing problems in our findings.

The cross-sectional study by Karanja et al. (44) on 177 children aged 6–12 years further confirms our results, highlighting that total emotional and behavioral symptoms were present in 46% of cases, mainly represented by attention problems, social problems, aggressive behavior, and withdrawal/depression.

Furthermore, a prospective controlled study of 43 preschool children with new onset epilepsy showed an increase in internalizing, externalizing, and total problems compared to controls both at the baseline and after 1 year of follow-up, suggesting the need to reassess these symptoms over time (45).

On the other hand, this study seemed to disagree with Cianchetti and colleagues (46) who found lower rates of anxiety (8%) and depression (9.2%) than ours in a sample of 326 children aged between 8 and 18 years. Probably, this discrepancy may be due to the different standardized neuropsychological tool, which was based on the self-assessment of child.

In this study, higher presence of total problems and internalizing problems was related to an early age of epilepsy onset, in keeping with previous studies (43, 47, 48).

The presence of emotional and behavioral problems in this study was not related to other sociodemographic variables. This finding disagreed with several literature studies, which report a significant association with age, duration of epilepsy, seizure frequency, number of ASMs, socioeconomic status, and family history of psychiatric disorders (41, 44, 45, 48–50). Possibly, our result could be attributed to the sample size that did not allow us to reach statistical significance. A future study on a larger sample will be needed.

The study by Moreira et al. (51), however, suggested no relationship between emotional and behavioral problems and other clinical variables (duration of epilepsy or number of ASMs), but highlighted a significant relationship with children IQ. It would, therefore, be interesting, in a future research, to add this parameter in our analysis.

The analysis of the PSI questionnaire detected clinical levels of total parental stress in 50% of the parents of children and adolescents with epilepsy. Clinical stress levels were detectable in the following subscales: PD (45%), P-CDI (57%), and DC (49%).

The statistical comparison showed significantly higher levels of parental stress in the parents of the group with epilepsy than in the parents of the control group.

This data are in agreement with previous studies, showing that parents of children with epilepsy experience significantly higher stress levels than general population (52, 53).

A 10-year longitudinal study that considered 356 mothers of children with epilepsy showed that 57% scored in the “at-risk” range for major depression. A supportive family environment was significantly associated with a better trend over time. Other significant factors were: seizure frequency, cognitive level of child, maternal age, and educational level (54).

The recent study by Olagunju et al. (55) showed that the perceived level of burden in 121 caregivers of adolescents with epilepsy (cases) was significantly higher than the one in caregivers of adolescents with sickle cell anemia (controls). In the cases group, significant levels of psychological parental distress were found in 38% and significant levels of depression/anxiety were found in 39.7%.

High levels of parental stress can be explained by several factors: worries of parent about the occurrence of future seizures, possible side effects of ASMs, social stigma, and lifestyle consequences of the disease.

In our previous studies, parental stress seemed to be unrelated to the severity of epilepsy (56) and seemed to persist even after therapy withdrawal (57). Moreover, children with severe epilepsy can also present behavioral, mood, and sleep disorders, which, in turn, contribute to increase stress in parents (57).

Another significant result of this study was that parental stress levels were significantly related to emotional and behavioral symptoms in their child, involving both the internalizing and externalizing problems. On the other hand, study by van den Berg and colleagues (58) showed that only externalizing problems were related to parental stress.

We can assume that externalizing problems, such as aggressive behaviors, can lead to a difficult child management, resulting in a feeling of inadequacy of the parents. The presence of internalizing problems, such as mood disorders and anxiety disorders, on the other hand, can increase the concern about the health of child, resulting in a parent–child dysfunctional interaction (59). Individual psychological factors of the caregivers can influence the impact of epilepsy in family life and the parental stress levels (60).

The strength of this study was the recruitment of an age-matched control group and the assessment through standardized neuropsychological tests. This study had certainly many limitations such as the modest sample size and the cross-sectional design.

In conclusion, children and adolescents with epilepsy are at a higher risk of developing emotional and behavioral problems such as anxiety, depression, somatic problems, and attention problems than their peers. The presence of emotional and behavioral problems can affect the parental stress and the quality of life of the entire family.

It is important to precociously detect emotional and behavioral symptoms in children with epilepsy in order to prevent the development of future psychopathological conditions (61, 62) and support their parents by providing them with adequate coping strategies (63–65).

## Data Availability Statement

The raw data supporting the conclusions of this article will be made available by the authors, without undue reservation.

## Ethics Statement

The studies involving human participants were reviewed and approved by Campania Sud Ethics Committee. Written informed consent to participate in this study was provided by the participants' legal guardian/next of kin.

## Author Contributions

FO and GP conceptualized the work. GP and FP analyzed the data and drafted the manuscript. CP, VV, and CS performed psychometric measurements and analyzed the data. IP revised English language and researched the data literature. GC was involved in planning and supervised the work. All authors have agreed to this final version and participated in a meaningful way in the preparation of the manuscript.

## Conflict of Interest

The authors declare that the research was conducted in the absence of any commercial or financial relationships that could be construed as a potential conflict of interest.

## Publisher's Note

All claims expressed in this article are solely those of the authors and do not necessarily represent those of their affiliated organizations, or those of the publisher, the editors and the reviewers. Any product that may be evaluated in this article, or claim that may be made by its manufacturer, is not guaranteed or endorsed by the publisher.

## References

[1] Guerrini R. Epilepsy in children. Lancet. (2006) 367:499–524. 10.1016/S0140-6736(06)68182-8 16473127

[2] Ettinger AB Weisbrot DM Nolan EE Gadow KD Vitale SA Andriola MR. Symptoms of depression and anxiety in pediatric epilepsy patients. Epilepsia. (1998) 39:595–9. 10.1111/j.1528-1157.1998.tb01427.x 9637601

[3] Ekinci O Titus JB Rodopman AA Berkem M Trevathan E. Depression and anxiety in children and adolescents with epilepsy: prevalence, risk factors, and treatment. Epilepsy Behav. (2009) 14:8–18. 10.1016/j.yebeh.2008.08.015 18804186

[4] Reilly C Agnew R Neville BG. Depression and anxiety in childhood epilepsy: a review. Seizure. (2011) 20:589–97. 10.1016/j.seizure.2011.06.004 21741277

[5] Verrotti A Carrozzino D Milioni M Minna M Fulcheri M. Epilepsy and its main psychiatric comorbidities in adults and children. J Neurol Sci. (2014) 343:23–9. 10.1016/j.jns.2014.05.043 24929650

[6] Polanczyk GV Salum GA Sugaya LS Caye A Rohde LA. Annual research review: a meta-analysis of the worldwide prevalence of mental disorders in children and adolescents. J Child Psychol Psychiatry. (2015) 56:345–65. 10.1111/jcpp.12381 25649325

[7] Pellock JM. Defining the problem: psychiatric and behavioral comorbidity in children and adolescents with epilepsy. Epilepsy Behav. (2004) 5:S3–9. 10.1016/j.yebeh.2004.06.010 15351340

[8] Dunn DW Austin JK Perkins SM. Prevalence of psychopathology in childhood epilepsy: categorical and dimensional measures. Dev Med Child Neurol. (2009) 51:364–72. 10.1111/j.1469-8749.2008.03172.x 19018836 PMC2860735

[9] Serra-Pinheiro MA D'andrea-Meira I Angelim AIM Fonseca FA Zimmermann N. High prevalence of psychiatric comorbidities in children and adolescents at a tertiary epilepsy center. Arq Neuropsiquiatr. (2021) 79:521–6. 10.1590/0004-282x-anp-2020-0202 34320056 PMC9394578

[10] Mula M Kanner AM Jetté N Sander JW. Psychiatric comorbidities in people with epilepsy. Neurol Clin Pract. (2021) 11:e112–20. 10.1212/CPJ.0000000000000874 33842079 PMC8032418

[11] Thome-Souza S Kuczynski E Assumpção F Jr Rzezak P Fuentes D Fiore L. Which factors may play a pivotal role on determining the type of psychiatric disorder in children and adolescents with epilepsy? Epilepsy Behav. (2004) 5:988–94. 10.1016/j.yebeh.2004.09.001 15582849

[12] Otero S. Psychopathology and psychological adjustment in children and adolescents with epilepsy. World J Pediatr. (2009) 5:12–7. 10.1007/s12519-009-0002-9 19172326

[13] Baki O Erdogan A Kantarci O Akisik G Kayaalp L Yalcinkaya C. Anxiety and depression in children with epilepsy and their mothers. Epilepsy Behav. (2004) 5:958–64. 10.1016/j.yebeh.2004.08.016 15582845

[14] Williams J Steel C Sharp GB DelosReyes E Phillips T Bates S. Anxiety in children with epilepsy. Epilepsy Behav. (2003) 4:729–32. 10.1016/j.yebeh.2003.08.032 14698708

[15] Davies S Heyman I Goodman R. A population survey of mental health problems in children with epilepsy. Dev Med Child Neurol. (2003) 45:292–5. 10.1017/S0012162203000550 12729141

[16] Kwong KL Lam D Tsui S Ngan M Tsang B Lai TS. Anxiety and depression in adolescents with epilepsy. J Child Neurol. (2016) 31:203–10. 10.1177/0883073815587942 26033229

[17] Aaberg KM Bakken IJ Lossius MI Lund Søraas C Håberg SE Stoltenberg C. Comorbidity and childhood epilepsy: a nationwide registry study. Pediatrics. (2016) 138:e20160921. 10.1542/peds.2016-0921 27482059

[18] Puka K Widjaja E Smith ML. The influence of patient, caregiver, and family factors on symptoms of anxiety and depression in children and adolescents with intractable epilepsy. Epilepsy Behav. (2017) 67:45–50. 10.1016/j.yebeh.2016.12.011 28088680

[19] Kanner AM. Psychiatric issues in epilepsy: the complex relation of mood, anxiety disorders, and epilepsy. Epilepsy Behav. (2009) 15:83–7. 10.1016/j.yebeh.2009.02.034 19245845

[20] Ben-Ari Y. Basic developmental rules and their implications for epilepsy in the immature brain. Epileptic Disord. (2006) 8:91–102. 16793570

[21] Verrotti A Cusmai R Laino D Carotenuto M Esposito M Falsaperla R. Long-term outcome of epilepsy in patients with Prader-Willi syndrome. J Neurol. (2015) 262:116–23. 10.1007/s00415-014-7542-1 25326049

[22] Operto FF Pastorino GMG Mazza R Roccella M Carotenuto M Margari L. Cognitive profile in BECTS treated with levetiracetam: a 2-year follow-up. Epilepsy Behav. (2019) 97:187–91. 10.1016/j.yebeh.2019.05.046 31252277

[23] Germanò E Gagliano A Arena C Cedro C Vetri L Operto FF. Reading-writing disorder in children with idiopathic epilepsy. Epilepsy Behav. (2020) 111:107118. 10.1016/j.yebeh.2020.107118 32563891

[24] Operto FF Pastorino GMG Mazza R Di Bonaventura C Marotta R Pastorino N. Social cognition and executive functions in children and adolescents with focal epilepsy. Eur J Paediatr Neurol. (2020) 28:167–75. 10.1016/j.ejpn.2020.06.019 32718867

[25] Pastorino GMG Operto FF Padovano C Vivenzio V Scuoppo C Pastorino N. Social cognition in neurodevelopmental disorders and epilepsy. Front Neurol. (2021) 12:658823. 10.3389/fneur.2021.658823 33935956 PMC8079621

[26] Villanueva V Girón JM Martín J Hernández-Pastor LJ Lahuerta J Doz M. Quality of life and economic impact of refractory epilepsy in Spain: the ESPERA study. Neurologia. (2013) 28:195–204. 10.1016/j.nrleng.2012.04.013 22743210

[27] Operto FF Pastorino GMG Mazza R Carotenuto M Roccella M Marotta R. Effects on executive functions of antiepileptic monotherapy in pediatric age. Epilepsy Behav. (2020) 102:106648. 10.1016/j.yebeh.2019.106648 31715510

[28] Operto FF Pastorino GMG Mazza R Di Bonaventura C Matricardi S Verrotti A. Perampanel tolerability in children and adolescents with focal epilepsy: effects on behavior and executive functions. Epilepsy Behav. (2020) 103:106879. 10.1016/j.yebeh.2019.106879 31937512

[29] Operto FF Verrotti A Marrelli A Ciuffini R Coppola G Pastorino GMG. Cognitive, adaptive, and behavioral effects of adjunctive rufinamide in Lennox-Gastaut syndrome: a prospective observational clinical study. Epilepsy Behav. (2020) 112:107445. 10.1016/j.yebeh.2020.107445 32920379

[30] Operto FF Vivenzio V Scuoppo C Padovano C Roccella M Quatrosi G. Perampanel and visuospatial skills in children with epilepsy. Front Neurol. (2021) 12:696946. 10.3389/fneur.2021.696946 34305800 PMC8296464

[31] Cianchetti C Messina P Pupillo E Crichiutti G Baglietto MG Veggiotti P. The perceived burden of epilepsy: impact on the quality of life of children and adolescents and their families. Seizure. (2015) 24:93–101. 10.1016/j.seizure.2014.09.003 25264356

[32] Cousino MK Hazen AR. Parenting stress among caregivers of children with chronic illness: a systematic review. J Pediatr Psychol. (2013) 38:809–28. 10.1093/jpepsy/jst049 23843630

[33] Craig F Operto FF De Giacomo A Margari L Frolli A Conson M. Parenting stress among parents of children with neurodevelopmental disorders. Psychiatry Res. (2016) 242:121–9. 10.1016/j.psychres.2016.05.016 27280521

[34] Fisher RS Cross JH French JA Higurashi N Hirsch E Jansen FE. Operational classification of seizure types by the international league against epilepsy: position paper of the ILAE commission for classification and terminology. Epilepsia. (2017) 58:522–30. 10.1111/epi.13670 28276060

[35] Achenbach TM Rescorla LA. Manual for the ASEBA School-Age Forms & Profiles. Burlington, VT: University of Vermont, Research Center for Children, Youth, & Families. Italian version: d'Orlando F, Grassi M, Di Blasi L. (2010) Giornale Italiano di Psicologia (2001). 37:919–43.

[36] Abidin RR. PSI-4: Parenting Stress Index-Fourth Edition. Lutz, FL: Psychological Assessment Resources.

[37] Guarino A Laghi F Serantoni G Di Blasio P Camisasca E. (2016). Parenting Stress Index – Fourth Edition (PSI-4), Firenze: Giunti O.S.

[38] Roeder R Roeder K Asano E Chugani HT. Depression and mental health help-seeking behaviors in a predominantly African American population of children and adolescents with epilepsy. Epilepsia. (2009) 50:1943–52. 10.1111/j.1528-1167.2009.02046.x 19260941

[39] Dagar A Falcone T. Psychiatric comorbidities in pediatric epilepsy. Curr Psychiatry Rep. (2020) 22:77. 10.1007/s11920-020-01195-8 33128638

[40] Scott AJ Sharpe L Loomes M Gandy M. Systematic review and meta-analysis of anxiety and depression in youth with epilepsy. J Pediatr Psychol. (2020) 45:133–44. 10.1093/jpepsy/jsz099 31904859

[41] Shehata N Saleh SM Kamal AM Awad OK. Assessment of the frequency of depressive symptoms in epileptic children (Single Center Study). Risk Manag Healthc Policy. (2021) 14:2089–97. 10.2147/RMHP.S301058 34295198 PMC8290486

[42] Jones JE Watson R Sheth R Caplan R Koehn M Seidenberg M. Psychiatric comorbidity in children with new onset epilepsy. Dev Med Child Neurol. (2007) 49:493–7. 10.1111/j.1469-8749.2007.00493.x 17593119

[43] Dal Canto G Pellacani S Valvo G Masi G Ferrari AR Sicca F. Internalizing and externalizing symptoms in preschool and school-aged children with epilepsy: focus on clinical and EEG features. Epilepsy Behav. (2018) 79:68–74. 10.1016/j.yebeh.2017.10.004 29253677

[44] Karanja SW Kiburi SK Kang'ethe R Othieno CJ. Emotional and behavioral problems in children with epilepsy attending the pediatric neurology clinic at a referral hospital in Kenya. Epilepsy Behav. (2021) 114:107477. 10.1016/j.yebeh.2020.107477 33288402

[45] Çelen Yoldaş T Günbey C Degerliyurt A Erol N Özmert E Yalnizoglu D. Behavioral problems of preschool children with new-onset epilepsy and one-year follow-up - a prospective study. Epilepsy Behav. (2019) 92:171–5. 10.1016/j.yebeh.2018.12.025 30660968

[46] Cianchetti C Bianchi E Guerrini R Baglietto MG Briguglio M Cappelletti S. Symptoms of anxiety and depression and family's quality of life in children and adolescents with epilepsy. Epilepsy Behav. (2018) 79:146–53. 10.1016/j.yebeh.2017.11.030 29289902

[47] Operto FF Pastorino GMG Marciano J de Simone V Volini AP Olivieri M. Digital devices use and language skills in children between 8 and 36 month. Brain Sci. (2020) 10:656. 10.3390/brainsci10090656 32967331 PMC7563257

[48] Mishra OP Upadhyay A Prasad R Upadhyay SK Piplani SK. Behavioral problems in Indian children with epilepsy. Indian Pediatr. (2017) 54:116–20. 10.1007/s13312-017-1012-7 28031547

[49] Oguz A Kurul S Dirik E. Relationship of epilepsy-related factors to anxiety and depression scores in epileptic children. J Child Neurol. (2002) 17:37–40. 10.1177/088307380201700109 11913568

[50] Caplan R Siddarth P Gurbani S Hanson R Sankar R Shields WD. Depression and anxiety disorders in pediatric epilepsy. Epilepsia. (2005) 46:720–30. 10.1111/j.1528-1167.2005.43604.x 15857439

[51] Moreira Fde S de Lima AB Fonseca PC Maia-Filho Hde S. Mental health of children and adolescents with epilepsy: analysis of clinical and neuropsichological aspects. Arq Neuropsiquiatr. (2014) 72:613–8. 10.1590/0004-282X20140098 25003397

[52] Cushner-Weinstein S Dassoulas K Salpekar JA Henderson SE Pearl PL Gaillard WD. Parenting stress and childhood epilepsy: the impact of depression, learning, and seizure-related factors. Epilepsy Behav. (2008) 13:109–14. 10.1016/j.yebeh.2008.03.010 18442950

[53] Klotz KA Özcan J Sag Y Schönberger J Kaier K Jacobs J. Anxiety of families after first unprovoked or first febrile seizure - a prospective, randomized pilot study. Epilepsy Behav. (2021) 122:108120. 10.1016/j.yebeh.2021.108120 34144460

[54] Puka K Ferro MA Anderson KK Speechley KN. Prevalence and trajectories of depressive symptoms among mothers of children with newly diagnosed epilepsy: a longitudinal 10-year study. Epilepsia. (2019) 60:358–66. 10.1111/epi.14638 30645767

[55] Olagunju AT Bioku AA Ohaeri JU Oluwaniyi SO Li A Olagunju TO. A comparative study of perceived burden in parent caregivers of adolescents with epilepsy in a resource-restricted setting: Investigating the explanatory factors of perceived burden. Epilepsy Behav. (2021) 120:107992. 10.1016/j.yebeh.2021.107992 33962249

[56] Operto FF Mazza R Pastorino GMG Campanozzi S Verrotti A Coppola G. Parental stress in a sample of children with epilepsy. Acta Neurol Scand. (2019) 140:87–92. 10.1111/ane.13106 31002402

[57] Operto FF Mazza R Pastorino GMG Campanozzi S Margari L Coppola G. Parental stress in pediatric epilepsy after therapy withdrawal. Epilepsy Behav. (2019) 94:239–42. 10.1016/j.yebeh.2019.03.029 30978636

[58] van den Berg L de Weerd AW Reuvekamp HF van der Meere JJ. The burden of parenting children with frontal lobe epilepsy. Epilepsy Behav. (2019) 97:269–74. 10.1016/j.yebeh.2019.05.034 31254848

[59] Operto FF Smirni D Scuoppo C Padovano C Vivenzio V Quatrosi G. Neuropsychological profile, emotional/behavioral problems, and parental stress in children with neurodevelopmental disorders. Brain Sci. (2021) 11:584. 10.3390/brainsci11050584 33946388 PMC8146823

[60] Jakobsen AV Elklit A. Self-control and coping responses are mediating factors between child behavior difficulties and parental stress and family impact in caregivers of children with severe epilepsy. Epilepsy Behav. (2021) 122:108224. 10.1016/j.yebeh.2021.108224 34352665

[61] Dreier JW Pedersen CB Cotsapas C Christensen J. Childhood seizures and risk of psychiatric disorders in adolescence and early adulthood: a Danish nationwide cohort study. Lancet Child Adolesc Health. (2019) 3:99–108. 10.1016/S2352-4642(18)30351-1 30528754 PMC6903917

[62] Operto FF Matricardi S Pastorino GMG Verrotti A Coppola G. The ketogenic diet for the treatment of mood disorders in comorbidity with epilepsy in children and adolescents. Front Pharmacol. (2020) 11:578396. 10.3389/fphar.2020.578396 33381032 PMC7768824

[63] Reilly C Atkinson P Memon A Jones C Dabydeen L Das KB. Symptoms of depression, anxiety, and stress in parents of young children with epilepsy: a case controlled population-based study. Epilepsy Behav. (2018) 80:177–83. 10.1016/j.yebeh.2017.12.020 29414549

[64] Bagherian B Nematollahi M Mehdipour-Rabori R. How parents cope with the care of a child with epilepsy: based upon grounded theory. Ethiop J Health Sci. (2021) 31:329–38. 10.4314/ejhs.v31i2.16 34158785 PMC8188076

[65] Maya Kaye A. Pediatric epilepsy and psychoeducational interventions: a review of the literature. Epilepsy Behav. (2021). 121:108084. 10.1016/j.yebeh.2021.108084 34107404

